# Cancer Hospital to Be Established in New York

**Published:** 1914-05

**Authors:** 


Cancer Hospital to Be Established in New York.—It is announced that plans are being prepared for the establishment in New York of an institution to be devoted to research into the causes and treatment of cancer. The General Memorial Hospital will be used for the exclusive treatment of cancer patients and the work will be under the control of Cornell Medical College. More than $1,000,000 is already in hand, in addition to the hospital buildings, and the institution will also have a large supply of radium. Dr. James Douglas, of New York, has not only donated $500,000 toward the fund, but has turned over to the hospital his half interest in the radium mines of Colorado, owned jointly by him and Dr. Howard A. Kelly, of Baltimore. It is said that it is largely due to Doctor Douglas's generosity that the establishment of the new institution has been made possible. The hospital will be under the direction of Dr. William M. Polk, dean of the medical college faculty, and Dr. James Ewing, professor of pathology. It is the intention of the new institution to take only those cases which are open to treatment or of interest in study.—*New York Medical Journal.*

